# The modulation on luminescence of Er^3+^-doped silicon-rich oxide films by the structure evolution of silicon nanoclusters

**DOI:** 10.1186/1556-276X-8-34

**Published:** 2013-01-18

**Authors:** Lu Jin, Dongsheng Li, Luelue Xiang, Feng Wang, Deren Yang, Duanlin Que

**Affiliations:** 1 State Key Laboratory of Silicon Materials and Department of Materials Science and Engineering, Zhejiang University, Hangzhou, 310027, People’s Republic of China

**Keywords:** Silicon-rich oxide, Silicon nanoclusters, Erbium ion, Energy transfer, Luminescence

## Abstract

A series of silicon-rich oxide (SRO) and erbium-doped SRO (SROEr) films imbedded with structural tunable silicon nanoclusters (Si NCs) have been fabricated using sputtering followed by post-annealing. The coalescence of Si NCs is found in the films with large Si excess. The energy transfer rate between Si NCs and Er^3+^ is enhanced, but the luminescence efficiencies of both Si NCs and Er^3+^ are reduced by the coalescent microstructures. Optimization of the microstructures of Si NCs is performed, and the preferential optical performance for both Si NCs and Er^3+^ could be achieved when Si NCs were separated in microstructures.

## Background

Er-doped silica-based materials have been extensively studied in the field of optical communication technology for their promising applications as active elements in photonic devices [1-4]. Indeed, the sharp luminescence of Er^3+^ ions at 1.54 μm matches the standard telecommunication wavelength of silica optical fibers and is absorption-free for Si bandgap. However, the Er^3+^ luminescence efficiency in silica is too low to be practical, and an expensive and bulky laser tuned to an Er^3+^ absorption band is required for the excitation of the Er^3+^ luminescence. Consequently, Si nanoclusters (Si NCs) with large excitation cross-section and broad excitation band are exploited as sensitizers to improve the excitation efficiency of Er^3+^[5,6]. Great deals of researches have committed effort to improve the properties of sensitizers (Si NCs) and to enhance the luminescence efficiency of Er^3+^[7-9].

As for the Si NCs, both experimental and theoretical studies indicate that the microstructures, especially the interfaces of Si NCs, play an active role in their optoelectronic properties [10-12]. Furthermore, the optical properties of Si NCs would also be affected by the coalescence of Si NCs, which is universal in silicon-rich oxide (SRO) matrix with sufficient Si excess and long-time post-annealing process [13,14]. However, there still exist incomprehension and uncertainties regarding the influence of microstructures of Si NCs on the Er^3+^ optical properties despite of the extensive studies on the sensitization process of Si NCs for Er^3+^.

In this letter, we report on the effect of microstructure evolution of Si NCs on the Er-related luminescence in erbium-doped SRO (SROEr) films. We address in a conclusive way that the coalescence of Si NCs in microstructures would reduce the luminescence of Si NCs, which would further quench the luminescence of Er^3+^. These results reveal that separated Si NCs are needed to obtain efficient Er^3+^ luminescence.

## Methods

SRO (SROEr) films were deposited on *p*-type silicon substrates by the sputtering (co-sputtering) of a pure Si target or Er_2_O_3_ and Si targets in the plasma of Ar-diluted 1% O_2_ atmosphere, where the amount of Si excess and the Er concentration were modulated by varying the r.f. power from 80 to 160 W for Si and from 15 to 20 W for Er_2_O_3_, respectively. The samples with Si excesses of 11%, 36%, 58%, and 88%, and Er concentration of about 5×10^19^ at./cm^−3^ were selected for detailed research. The atomic compositions of the films were detected by Rutherford backscattering analysis using 2.02 MeV ^4^He ion beam at a scattering angle of 165°. The Si excess (*N*_Si-ex_) in this work can be calculated as *N*_Si-ex_ = (*N*_SRO_ − *N*_SiO2_)/*N*_SiO2_, where *N*_SRO_ and *N*_SiO2_ stand for the atomic percentage of Si atoms in SRO matrix and that in the SiO_2_ matrix, respectively. After the deposition of films, a thermal annealing procedure at 1,100°C for 1 h in a quartz furnace under the nitrogen ambient was performed to separate Si NCs and to activate Er ions. The structural characteristics of the films were studied by high-resolution transmission electron microscopy (HRTEM; Tecnai G2 F20 S-Twin microscope (FEI, Eindhoven, Netherlands)) cross-sectional images. Room temperature photoluminescence (PL) was measured at the same test conditions using He-Cd laser with the excitation wavelength of 325 nm and detected by charge-coupled device (PIXIS:100BR, Princeton Instruments, Trenton, America) or photomultiplier tube (Hamamatsu R5509-72, Hamamatsu Photonics K.K., Hamamatsu, Japan). For the time-resolved PL detected by a multichannel photon counting system (Edinburg Photonics, Livingston, UK), the samples were excited by a microsecond lamp with 325-nm line, and the overall time resolution of the system was about 2 μs.

## Results and discussion

**Figure 1:**
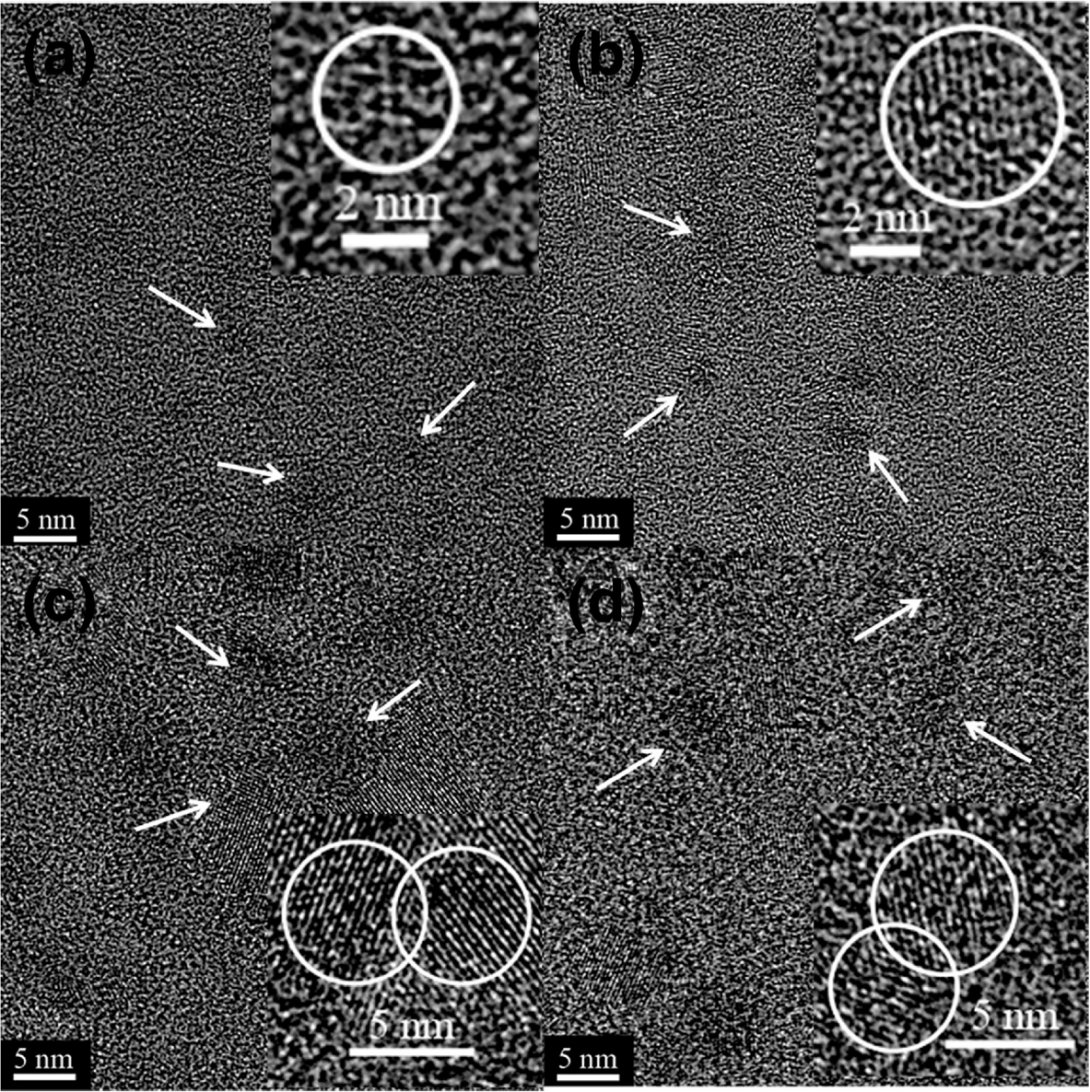
**HRTEM images of the SROEr films with different Si excesses. **(**a**) 11%, (**b**) 36%, (**c**) 58%, and (**d**) 88%. The Si NCs are indicated by white arrows. The insets display the HRTEM images of Si NCs in the SROEr films. The coalescent Si NCs can be formed in the SROEr films with high Si excess.

The influence of Si excess on the microstructures of Si NCs in SROEr films is studied using HRTEM, as shown in Figure 1. It can be seen that the size of Si NCs increases slightly from 2 to 5 nm in the films with the Si excess from 11% to 88%. The density of Si NCs (indicated by white arrows) is similar to each other in all these films (on the order of 10^12^ cm^−2^) except for that with the Si excess of 11%. Si NCs in the film with the Si excess of 11% exhibit much smaller sizes, which is under the resolution of the HRTEM. In this work, we assume that the Si NCs density is similar and has an insignificant influence on the luminescent property of the films. Furthermore, no Er^3+^ clusters are found in all the films so that the quenching phenomenon caused by Er^3+^ clustering could also be disregarded [15]. Interestingly, Si NCs are separately embedded in the matrix with lower Si excess, as shown in the inset of Figure 1a,b. In contrast, the coalescence of neighboring Si NCs is found in the films with higher Si excess (Figure 1c,d), which are caused by an asymptotic ripening process [16].

**Figure 2:**
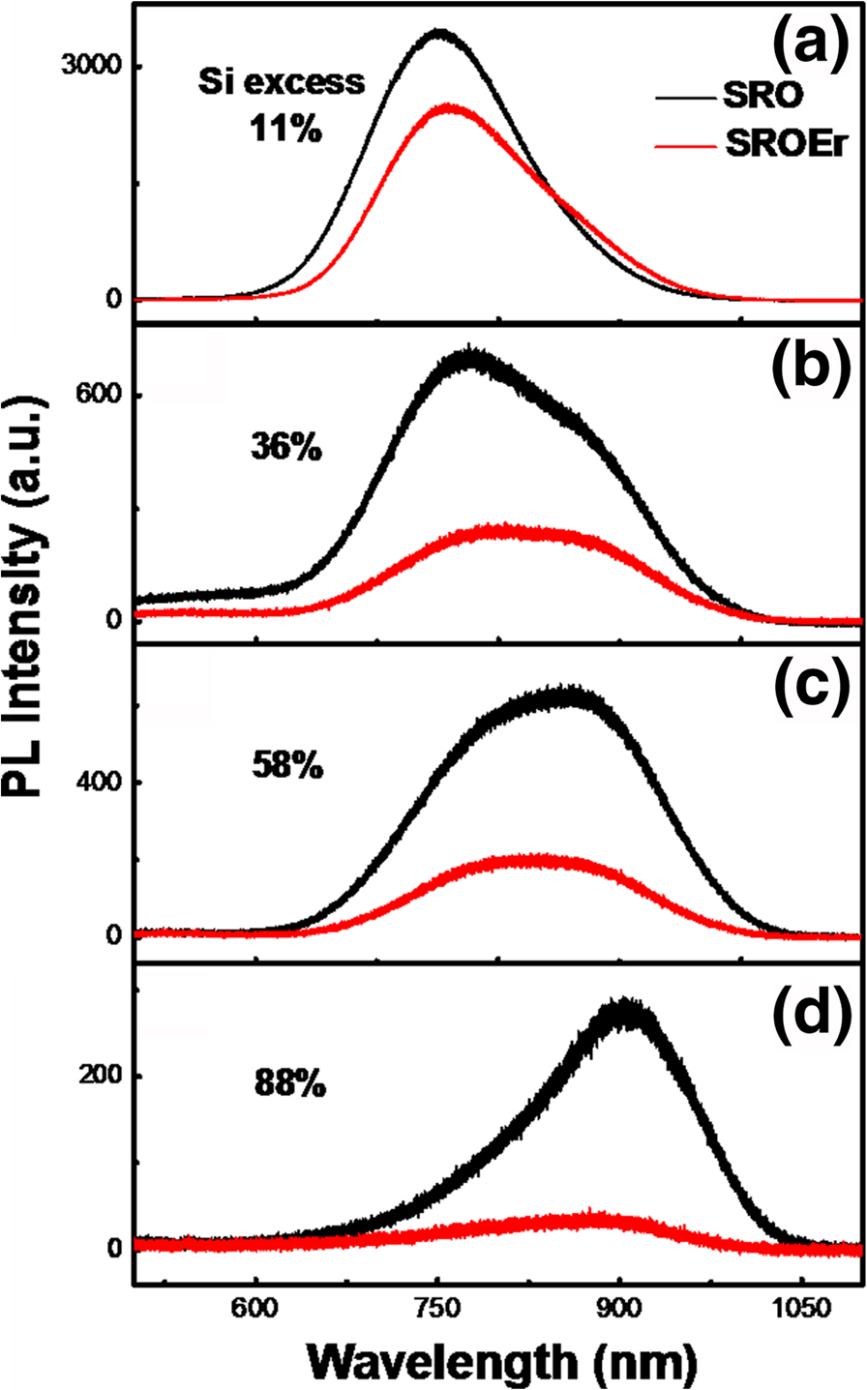
**Room-temperature PL spectra of Si NCs in the SRO and SROEr films.** The Si excesses in SRO and SROEr films are (**a**) 11%, (**b**) 36%, (**c**) 58%, and (**d**) 88%, respectively. The Si NCs with separated microstructures and smaller sizes might be preferable to their luminescence performance.

**Figure 3:**
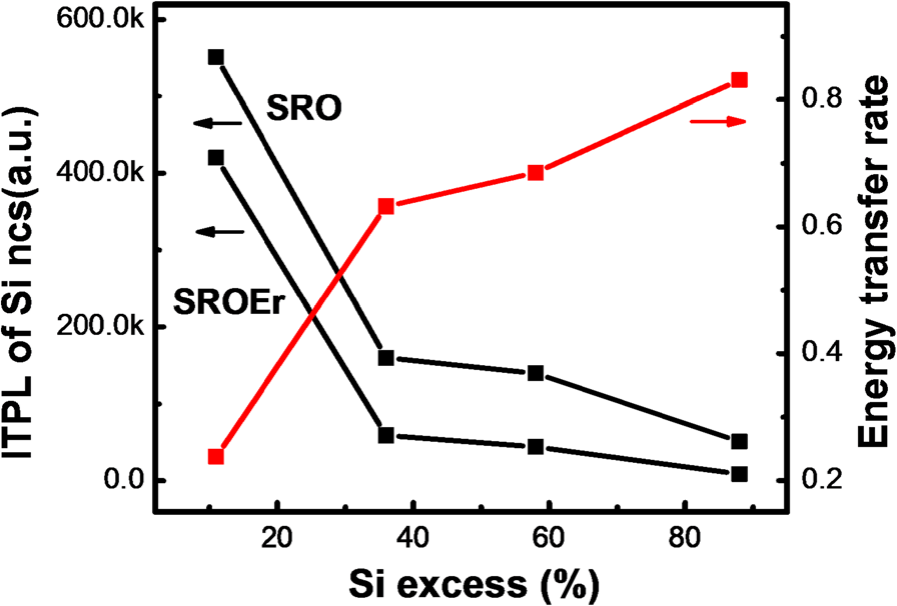
**ITPL intensity and energy transfer rate.** ITPL intensity of Si NCs in the SRO and SROEr films (left coordinate) and energy transfer rate between Si NCs and Er^3+^ (right coordinate) as a function of Si excesses. The energy transfer rate increases with the Si excess.

For the investigation of these Si NCs microstructural differences on the luminescence performance of the films, the PL spectra of the SRO and SROEr films with different Si excesses are provided, as shown in Figure 2. With the increase of the Si excess from 11% to 88%, the PL intensity of the Si NCs decreases, and its central wavelength red-shifts from 750 to 900 nm. One order of magnitude decrease of the integrated PL (ITPL) intensity can be observed by increasing the Si excess, as shown in Figure 3 (left axis). As we know, the redshift of PL central wavelength with the increase of Si excess as well as the size of Si NCs is mainly originated from the quantum confinement effect [17]. Furthermore, the lattice distortion in Si NCs and dangling bonds at defect centers could contribute to the decrease of PL intensity [18]. Therefore, the coalescence of Si NCs in the film with higher Si excess by asymptotic ripening process will deteriorate the microstructures (lattice distortion and dangling bonds) of Si NCs and then introduce more nonradiative recombination centers and interface states, resulting in the degeneration of the PL intensity of Si NCs, as shown in Figures 2 and 3. Moreover, the decrease of the exciton recombination rate in Si NCs with large size caused by the quantum confinement effect would also weaken their PL intensity. Consequently, the Si NCs with separated microstructures and smaller sizes might be preferable to their luminescence performance.

The evolution of the microstructures of Si NCs on the energy transfer process from Si NCs to the neighboring Er^3+^ ions is also checked. A distinct decrease of the PL intensity of Si NCs can be observed due to this energy transfer process [19], as shown in Figures 2 and 3. The efficiency of this energy transfer process can be characterized by the coupling efficiency (*η*) between Er^3+^ ions and Si NCs, which is expressed by the following [13]:

(1) $$\eta =\frac{\mathrm{ITP}L_{\mathrm{SRO}}-\mathrm{ITP}L_{\mathrm{SROEr}}}{\mathrm{ITP}L_{\mathrm{SRO}}}\text{,}$$

where ITPL_SRO_ and ITPL_SROEr_ are the integrated PL intensities of Si NCs in the SRO and SROEr films, respectively. As shown in Figure 3 (right axis), the *η* increases from 0.24 for the film with Si excess of 11% to 0.83 for that of 88%, while the coalescence of Si NCs is formed in films with large Si excess. The increase of energy transfer rate is partially caused by the more efficient sensitization capability of Si NCs with larger size due to their larger absorption cross-section [11]. Furthermore, the distance (*γ*) between Si NCs and Er^3+^ ions decreases with the increase in the size of Si NCs by assuming that the Er^3+^ ions are homogeneously distributed in the films from which the energy transfer rate is enhanced since the Förster energy transfer rate acts on a smaller *γ* scales as 1/*γ*^6^, while the far-field radiative interaction with a rate of 1/*γ*^2^ dominates for a larger *γ*[20].

**Figure 4:**
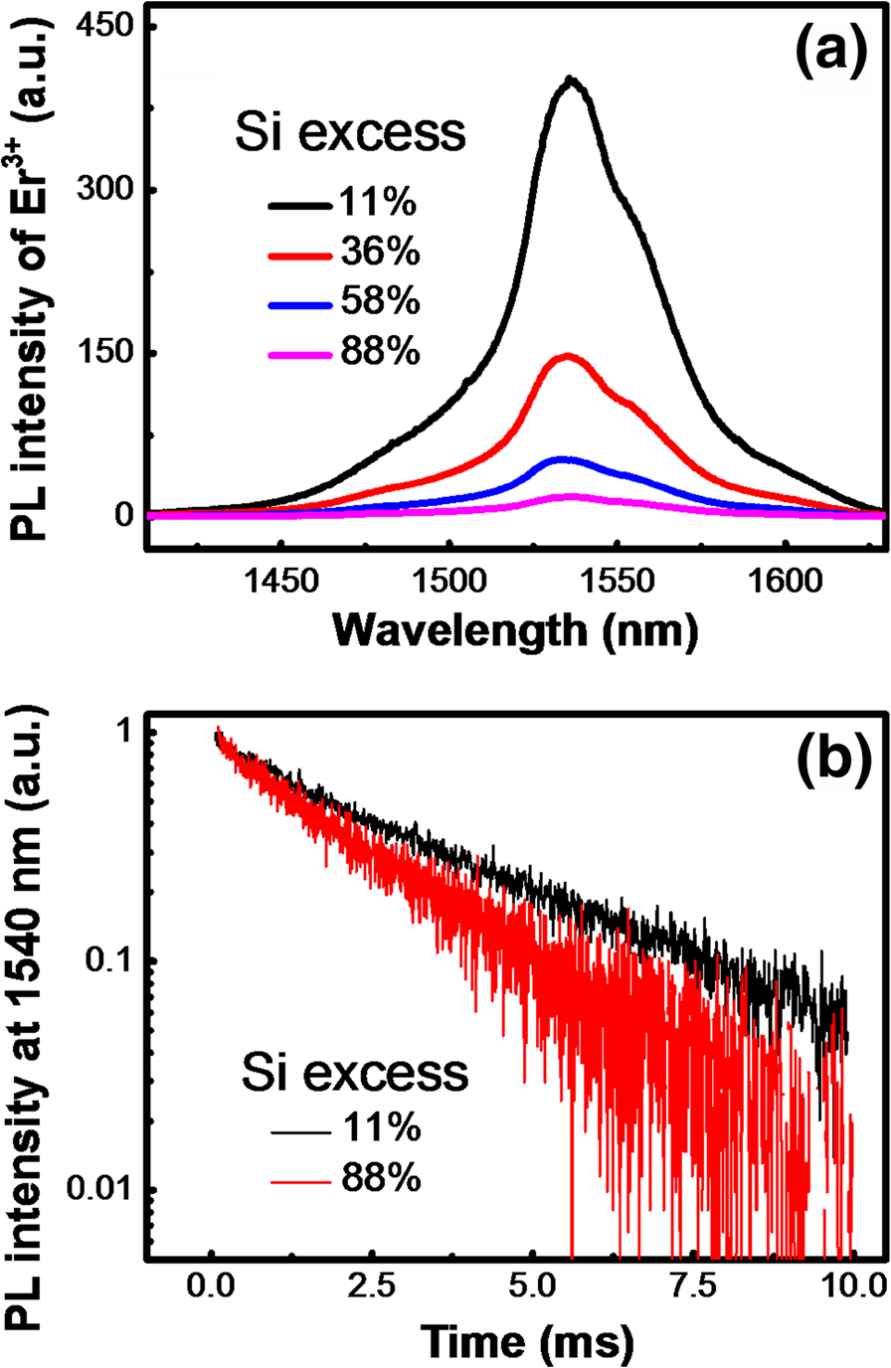
**Room-temperature PL spectra and decay curves of Er^3+^ion.** (**a**) Room-temperature PL spectra of Er^3+^ ion in the SROEr films. (**b**) Decay curves of PL signal recorded at 1,540 nm for Er^3+^ in SROEr films with Si excesses of 11% and 88%. Optimized Si NCs with separated microstructures are requested to obtain efficient Er^3+^ luminescence.

To determine the effect of this energy transfer process on the luminescence properties of Er^3+^ in the SROEr films with different Si NCs microstructures, the PL spectra of Er^3+^ in the films are provided, as shown in Figure 4a. Interestingly, the PL intensity of Er^3+^ decreases with the increase of the Si excesses, which is completely opposite to the evolution of the *η* but coincident with that of the original PL intensity of Si NCs, as shown in Figures 2 and 3. To further determine the effect of Si NCs microstructures on the transition between intra-4f levels of Er^3+^ ions (^4^I_13/2_ - ^4^I_15/2_), PL decay curves at the emission wavelength of Er^3+^ (1.54 μm) are provided, as shown in Figure 4b. From their fittings by stretched exponential function, we obtained that the characteristic decay time is on the order of millisecond (the curves of SROEr with the Si excess of 36% and 58% are not shown here). The largest value is obtained from the film with the lowest Si excess, which means that higher Si excess and the coalescence of Si NCs would enhance the nonradiative recombination of Er^3+^ ions. Nevertheless, the amount of Si excess has an insignificant effect on the luminescence performance of Er^3+^ as the variation of the characteristic decay time can be negligible, as shown in Figure 4b. Since the size and density of Si NCs for the sample with the Si excess of 36% were similar to the one with the Si excess of 88%, as shown in Figure 1b,d, while the PL intensity is significantly decreased, we ascribe the main origin of this decreased PL intensity as the microstructural differences of the Si NCs in these samples. Furthermore, the decrease of the oscillator strength with the increasing size of the Si NCs due to the coalescence might be also a partial reason for this decreased PL intensity. Besides, the influence of Si excess on the percentage of optically active Er^3+^ ions was also considered. Since the excitation energy in our experiment is especially low (about 3 × 10^16^ cm^−2^ s^−1^), the number of Er^3+^ ions contributing to the 1.5-μm emission could be assumed to be equal to the concentration of Si NCs acting as sensitizers [21]. Actually, Si NCs with similar densities have been obtained from SROEr films with different Si excesses in our experiment, as shown in Figure 1. It means that the influence of the percentage of optically active Er^3+^ on the luminescent property of the samples with different amounts of the Si excess is insignificant. Therefore, the microstructures of Si NCs play an extremely important role on the emission of Er^3+^ ions. The Si NCs with separated microstructures should be prepared for the further improvement of the luminescence performance of Er^3+^ ions.

## Conclusions

In summary, the effect of microstructure evolution of Si NCs on the Er-related luminescence has been investigated. The SRO and SROEr films were fabricated by sputtering. The structural and optical properties of the films are readily presented, and the coupling efficiency between Si NCs and Er^3+^ ions is studied. We found that while energy transfer process is more effective for coalescent Si NCs with larger sizes, the Er^3+^ luminescence efficiency is reduced by the spoiled microstructures of the sensitizer and the limited nonphonon recombination probability in large Si NCs. These results suggest that optimized Si NCs with separated and intact microstructures are requested to obtain efficient Er^3+^ luminescence.

## Abbreviations

HRTEM: High-resolution transmission electron microscopy; ITPL: Integrated photoluminescence; PL: Photoluminescence; Si NCs: Silicon nanoclusters; SRO: Silicon-rich oxide; SROEr: Erbium-doped silicon-rich oxide.

## Competing interests

The authors declare that they have no competing interests.

## Authors’ contributions

LJ performed the experiments, collected and analyzed the data, and wrote the paper; DL conceived the experiment, analyzed the results, and wrote the paper; LX, FW, DY and DQ helped with the data analysis and wrote the paper. All authors read and approved the final manuscript.

## Authors’ information

DL received his Ph.D. degree in the State Key Laboratory of Silicon Materials and Department of Material Science and Engineering from Zhejiang University, Hangzhou, China, in 2002. He is currently an associate professor in the Department of Material Science and Engineering at Zhejiang University. His current research interests include the synthesis of plasmonic microstructure, application of plasmonic microstructure on solar cells, Raman and luminescence, and silicon photonics. LJ, LX, and FW are currently the Ph.D. students in the State Key Laboratory of Silicon Materials and Department of Materials Science and Engineering, Zhejiang University, Hangzhou, China. Their current research interests include luminescence from erbium-doped silicon-rich oxide matrix, silicon-rich nitride matrix, and dislocations in silicon, silicon nitride-based light-emitting devices, and localized surface plasmon resonance of metal nanostructures. DY received his B.S. degree from Zhejiang University, Hangzhou, China, in 1985, and his Ph.D. degree in Semiconductor Materials from the State Key Laboratory of Silicon Materials in Zhejiang University, Hangzhou, China, in 1991. He has been with the Institute of Metal Materials in Tohoku University, Japan, and worked for Freiberg University, Germany, from 1995 to 1997. He is currently the director of the State Key Laboratory of Silicon Materials. His current research interests include the fabrication of single crystalline silicon materials for ultra-larger-scale integrated circuit and defect engineering, polysilicon materials and compound thin film photo-electric conversion materials for photovoltaic, nano-scale silicon wire/tube and other one-dimensional semiconductor materials, and silicon-based materials for optoelectronics. DQ received his B.S. degree in Department of Electrical Engineering from Xiamen University, Xiamen, China, in 1951. He has been with the Department of Electrical Engineering, Department of Radio-based Semiconductor Materials and Devices, Department of Materials Science and Engineering in Zhejiang University, China, since 1953.
